# Comparative analysis of liver cancer burden trends in China and worldwide between 1990 and 2021

**DOI:** 10.3389/fpubh.2025.1513210

**Published:** 2025-05-23

**Authors:** Chunrong Chen, Qianjie Xu, Tao Wang, Yuliang Yuan, Zuhai Hu, Xiaosheng Li, Xiaodong Zheng, Haike Lei

**Affiliations:** ^1^Department of Health Information Management, School of Public Health and Management, Chongqing Three Gorges Medical and Pharmaceutical College, Chongqing, China; ^2^Chongqing Cancer Multiomics Big Data Application Engineering Research Center, Chongqing University Cancer Hospital, Chongqing, China; ^3^Department of Health Statistics, School of Public Health, Chongqing Medical University, Chongqing, China; ^4^Affiliated Hospital of Chongqing Medical and Pharmaceutical College, Chenjiaqiao Hospital of Shapingba District, Chongqing, China

**Keywords:** liver cancer, global burden of disease study, China, epidemiology, trends analysis

## Abstract

**Background:**

The Global Burden of Diseases, Injuries, and Risk Factors Study (GBD) 2021 provides comprehensive data on liver cancer burden worldwide. This study aims to analyze and compare the trends in liver cancer burden in China and globally between 1990 and 2021.

**Methods:**

Data were extracted from the GBD 2021 database, which includes incidence, prevalence, mortality, and disability-adjusted life years (DALYs) for liver cancer in 204 countries and territories. Age-standardized rates (ASIR, ASPR, ASMR, ASDR) and crude rates (CIR, CPR, CMR, CDR) were calculated. Joinpoint regression analysis was used to determine the average annual percentage change (AAPC) in liver cancer burden trends.

**Results:**

In China, the number of liver cancer cases increased from 96,434 in 1990 to 196,637 in 2021, while the age-standardized incidence rate (ASIR) decreased. Globally, cases increased from 244,689 to 529,202, with a slight increase in ASIR. The age-standardized prevalence rate (ASPR) in China remained stable, while globally it increased. Age-standardized mortality rates (ASMR) and age-standardized DALY rates (ASDR) decreased in both China and globally. Males had higher rates than females in all age groups. The peak age for liver cancer burden occurred earlier in Chinese males compared to the global average.

**Conclusion:**

The liver cancer burden in China has declined over the past three decades, with a more significant decrease in China than globally. However, China still faces a higher burden compared to the global average. The earlier peak in liver cancer burden among Chinese males suggests the need for targeted prevention and control measures, especially in light of the impact of risk factors like hepatitis B and C, alcohol consumption, drug abuse, obesity, diabetes and tobacco use. Globally, liver cancer remains a significant public health challenge with rising incidence and prevalence rates, emphasizing the need for a comprehensive global approach to liver cancer prevention.

## Introduction

Malignant tumors have become one of the most severe non-communicable diseases threatening human health, constituting a significant global public health challenge (1). As one of the most common malignancies, liver cancer ranks sixth in incidence and third in mortality among all cancers (2), remaining a significant global health issue with a substantial disease burden. This burden poses a considerable threat to the health of populations worldwide, particularly in Asia and China. Approximately 75% of liver cancer cases occur in Asia, with China alone accounting for more than 50% of the global burden (3, 4). Studies have shown that the incidence, prevalence, mortality, and disability-adjusted life years (DALYs) of liver cancer in China are significantly greater than the global average and that the peak age at which the burden of liver cancer occurs earlier in both Chinese men and women (5, 6). Over the past few decades, the incidence of liver cancer has declined in many countries, particularly in previously high-burden Asian nations (7). However, an increasing trend has been observed in regions with historically low incidence rates, such as the United States (8, 9), highlighting the need for strengthened prevention and control measures against liver cancer. Therefore, utilizing the GBD 2021 database to compare and analyze the latest characteristics and trends of liver cancer burden in China and globally is essential for comprehending the potential implications of the COVID-19 pandemic on this disease burden.

On May 16, 2024, the Institute for Health Metrics and Evaluation (IHME) at the University of Washington updated the Global Burden of Diseases, Injuries, and Risk Factors Study (GBD) 2021 database (10). The GBD 2021 study investigated global health trends; revealed disparities related to age, sex, geography, and socioeconomic status; and highlighted the impact of the COVID-19 pandemic and other health challenges (7, 10–12). While global rates of overall disease burden declined by 14.2% between 2010 and 2019, the COVID-19 pandemic disrupted these declining trends, resulting in increases of 4.1% in 2020 and 7.2% in 2021 (10). Life expectancy declined in 84% of countries and territories during the COVID-19 pandemic, with a sharp global decrease of 1.6 years from 2019 to 2021, marking the largest drop in life expectancy of over 50 years (10). Despite the pandemic’s devastating effects, the global life expectancy increased by 22.7 years from 1950 to 2021, and child mortality rates have continued to decline throughout the pandemic (10). This study also explored demographic trends, including the global fertility transition, which will result in 97% of countries and territories falling below the replacement level of the fertility rate of 2.1 births per woman by 2,100, raising concerns about population aging (13, 14). Furthermore, GBD 2021 investigated the burden of non-communicable diseases and the impact of risk factors, such as high blood sugar, drug use, and obesity, on health outcomes (15). The study projects a global life expectancy increase of 4.6 years over the next three decades, with a decline in ill health and premature mortality due to communicable, maternal, neonatal, and nutritional diseases from 2022 to 2050, while the burden from non-communicable diseases is projected to rise (13, 14).

Therefore, as an important non-communicable disease, it is necessary to conduct research on the trends in disease burden of liver cancer using data from the GBD 2021 database.

## Methods

### Data source

All data used in this study were extracted from the Global Burden of Diseases, Injuries, and Risk Factors Study (GBD) 2021 database, which was updated on May 16, 2024, by the Institute for Health Metrics and Evaluation (IHME) at the University of Washington, Seattle, USA. The GBD 2021 provides comprehensive estimates of incidence, prevalence, mortality, disability-adjusted life years (DALYs), and 88 risk factors for 371 diseases and injuries across 204 countries and territories, including 811 subnational locations. The data are stratified by 25 age groups, ranging from birth to 95 years and older. Various sources, such as vital registration systems, verbal autopsies, censuses, household surveys, disease-specific registries, and health service contact data, are utilized in the GBD study. Additionally, the IHME incorporates data through systematic literature reviews.

The estimates of DALYs in the GBD 2021 study are derived from 100,983 data sources, of which 19,189 were newly used in 2021. Among these, 75,459 sources pertain to non-fatal causes, including 36,916 sources for incidence, 22,236 for prevalence, and 45 for other epidemiological metrics. Age and sex-specific data for China and globally were downloaded for the period 1990 to 2021 to assess the liver cancer burden. Since the GBD 2021 data are publicly available and intended solely for academic research, with no commercial use involved, ethical approval for data access and use was not required. The URL information of the data sources: https://vizhub.healthdata.org/gbd-results/.

### Statistical analysis

This study analyzed data on the incidence, prevalence, mortality, and DALYs of liver cancer in China and globally, key metrics included age-standardized incidence rates (ASIR), age-standardized prevalence rates (ASPR), age-standardized mortality rates (ASMR), and age-standardized DALY rates (ASDR). Additionally, crude incidence rates (CIR), crude prevalence rates (CPR), crude mortality rates (CMR), and crude DALY rates (CDR) were calculated for each age group.

To assess trends in liver cancer burden, joinpoint regression analysis was conducted using Joinpoint software (version 5.2.0; National Cancer Institute, Rockville, MD, United States). This method was used to calculate the average annual percentage change (AAPC) and corresponding 95% confidence intervals (CIs) (16, 17). The log-transformed age-standardized rates were modeled using the formula: ln(y) = *α* + *β*x + *ε*, where y represents the respective age-standardized rate and where x represents the calendar year (18). The AAPC was calculated as 100 × (exp(β) − 1) with the 95% CI derived from the model (18). An increasing trend was defined by an AAPC with a 95% CI greater than 0, a decreasing trend by a 95% CI less than 0, and a stable trend if the CI included 0 (18). Data analysis and visualization were performed using R statistical software (version 4.4.1) and Joinpoint software (version 5.2.0). A *p*-value of less than 0.05 was considered statistically significant.

## Results

### Characterization of the liver cancer burden in China and globally

**Figure 1:**
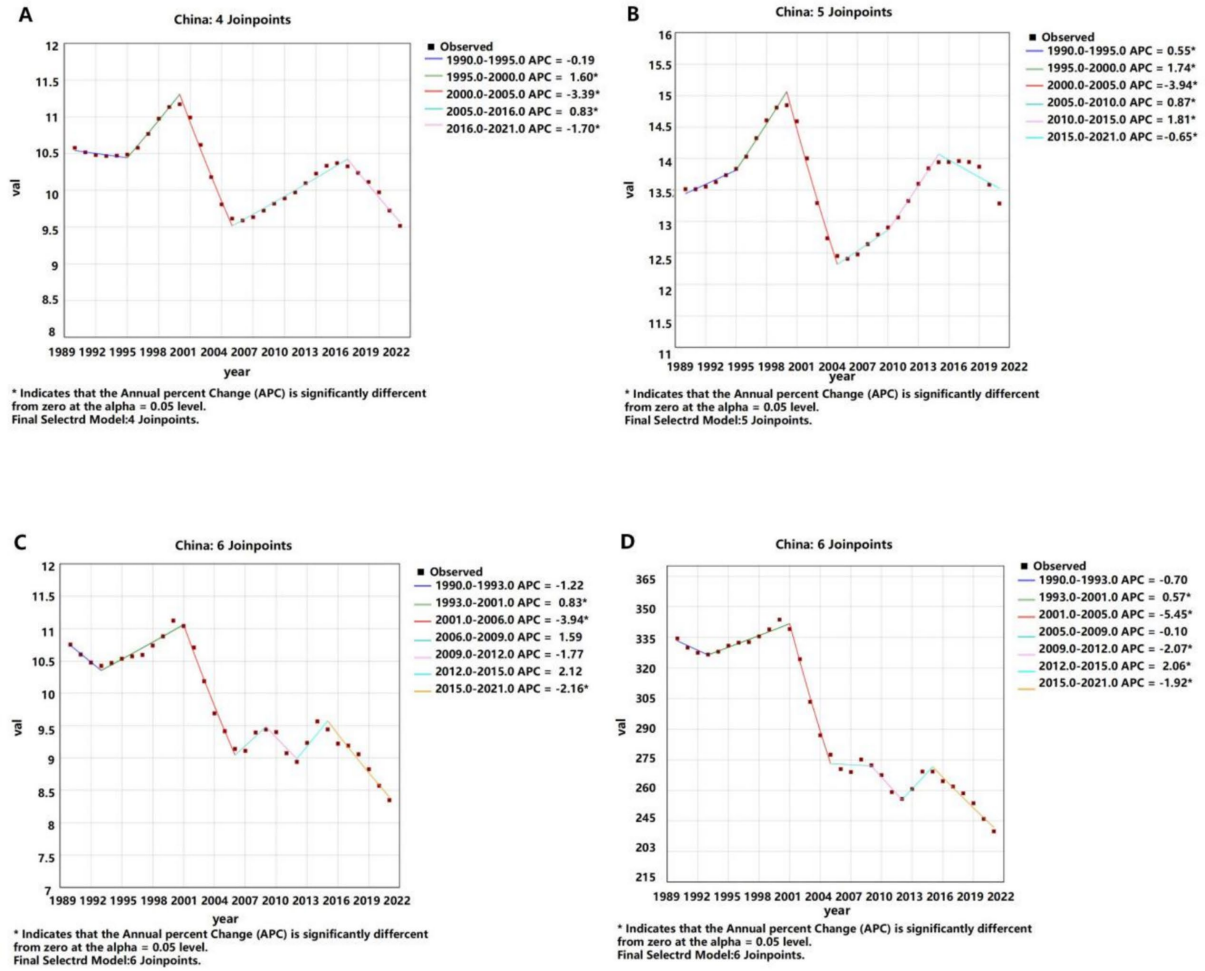
APCs of the ASIR, ASPR, ASMR, and ASDR of liver cancer in China from 1990 to 2021 (* indicates *p*-values < 0.05 and significant results). **(A)** ASIR; **(B)** ASPR; **(C)** ASMR; **(D)** ASDR.

**Figure 2:**
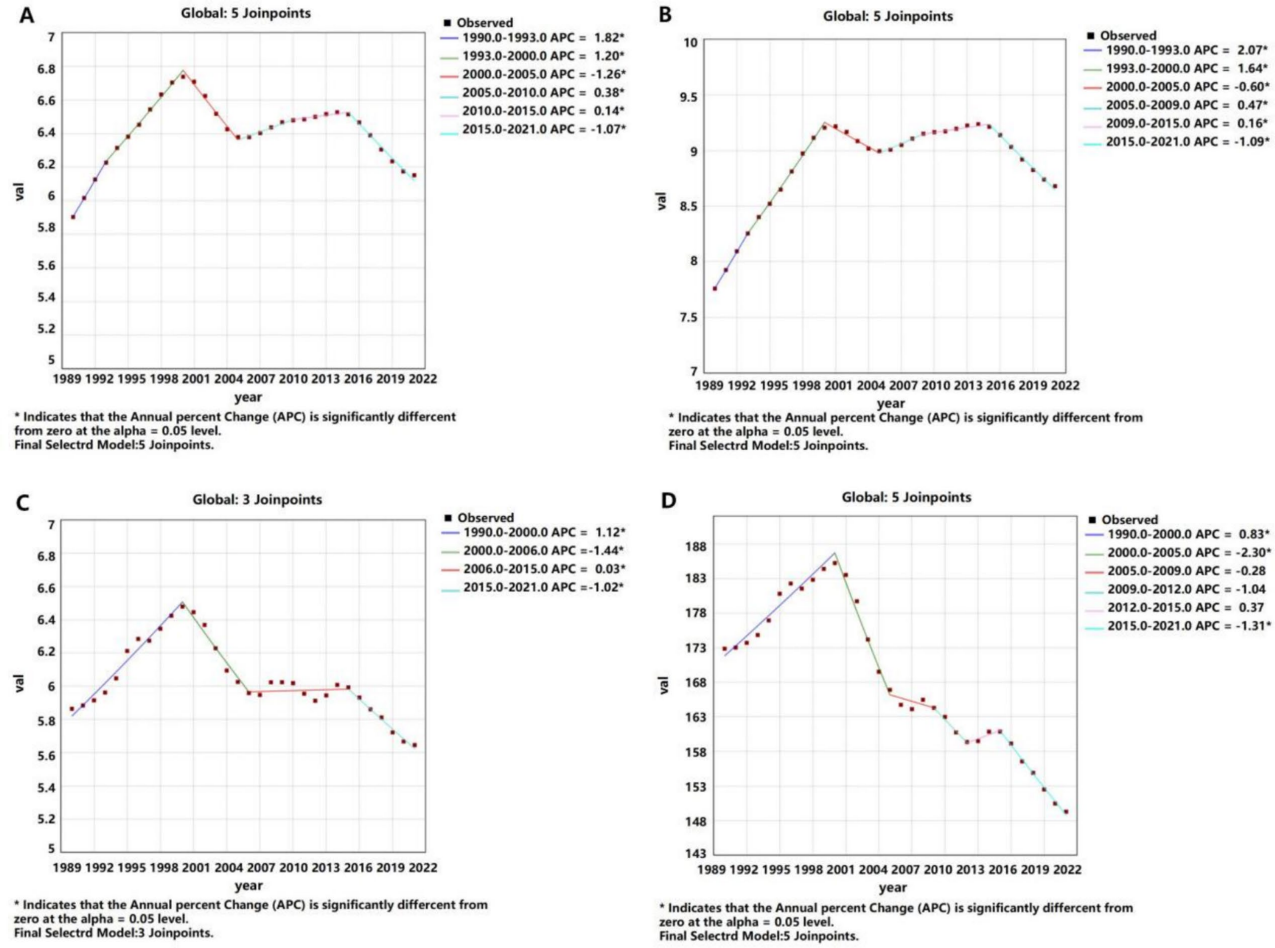
APCs of the ASIR, ASPR, ASMR, and ASDR of liver cancer worldwide from 1990 to 2021 (* indicates *p*-values < 0.05 and significant results). **(A)** ASIR; **(B)** ASPR; **(C)** ASMR; **(D)** ASDR.

**Figure 3:**
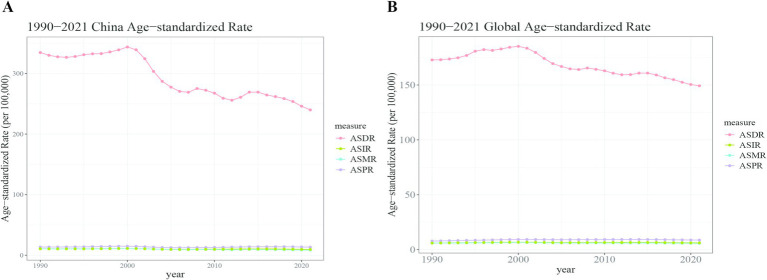
Trend comparison of the ASIR, ASPR, ASMR, and ASDR of liver cancer in China and worldwide from 1990 to 2021. **(A)** China; **(B)** Global.

In China, the number of liver cancer cases increased from 96,434 (95% CI: 80,971 – 113,769) in 1990 to 196,637 (95% CI: 158,273 – 243,558) in 2021. Despite this increase in absolute numbers, the ASIRs experienced a decline from 10.58 per 100,000 population (95% CI: 8.94–12.43) in 1990 to 9.52 per 100,000 population (95% CI: 7.72–11.78) in 2021, resulting in an average annual percentage change (AAPC) of −0.31% (95% CI: −0.39 to −0.23). Globally, liver cancer cases rose from 244,689 (95% CI: 224,795 – 268,549) to 529,202 (95% CI: 480,339 – 593,849) during the same period, with a slight increase in ASIR from 5.90 per 100,000 people (95% CI: 5.43–6.48) to 6.15 per 100,000 people (95% CI, 5.58–6.90), reflected by an AAPC of 0.11% (95% CI, 0.07–0.16) (Table 1 and Figures 1–3).

**Table 1:** All-age cases and age-standardized incidence, prevalence, mortality, and DALY rates and corresponding AAPCs in liver cancer in China and globally in 1990 and 2021.

Location	Measure	1990		2021		1990–2021 AAPC (%) *R* (95% CI)
		Number *n* (95% CI)	ASR (1/100,000) *R* (95% CI)	Number *n* (95% CI)	ASR (1/100,000) *R* (95% CI)	
China	Incidence	96,434 (80971–113,769)	10.58 (8.94–12.43)	196,637 (158273–243,558)	9.52 (7.72–11.78)	−0.31 (−0.39 – −0.23)
	Prevalence	132,779 (108924–155,564)	13.52 (11.20–15.78)	265,539 (212435–331,149)	13.29 (10.75–16.41)	0.02 (−0.11–0.15)
	Deaths	94,937 (79884–111,527)	10.75 (9.12–12.61)	172,068 (139621–212,496)	8.35 (6.80–10.29)	−0.79 (−1.18 – −0.41)
	DALYs	3,294,864 (2763029–3,879,589)	334.52 (281.08–393.14)	4,890,023 (3905089–6,124,599)	239.91 (191.98–299.37)	−1.03 (−1.32 – −0.74)
Global	Incidence	244,689 (224795–268,549)	5.90 (5.43–6.48)	529,202 (480339–593,849)	6.15 (5.58–6.90)	0.11 (0.07–0.16)
	Prevalence	345,913 (299827–376,632)	7.76 (6.92–8.43)	739,300 (673114–821,948)	8.68 (7.91–9.67)	0.35 (0.31–0.39)
	Deaths	238,969 (218717–263,037)	5.86 (5.38–6.46)	483,875 (440400–540,177)	5.65 (5.13–6.30)	−0.11 (−0.23–0.01)
	DALYs	7,553,667 (6897511–8,296,182)	172.86 (157.84–190.16)	12,887,652 (11673533–14,472,228)	149.29 (135.24–167.49)	−0.46 (−0.67 – −0.26)

In terms of prevalence, the number of liver cancer cases in China more than doubled, increasing from 132,779 (95% CI: 108,924 – 155,564) in 1990 to 265,539 (95% CI: 212,435 – 331,149) in 2021. However, the ASPR remained relatively stable, with a slight decrease from 13.52 per 100,000 (95% CI: 11.20–15.78) to 13.29 per 100,000 (95% CI: 10.75–16.41), reflected by an AAPC of 0.02% (95% CI: −0.11 – 0.15). Globally, the ASPR increased from 7.76 per 100,000 in 1990 to 8.68 per 100,000 in 2021, reflected by an AAPC of 0.35% (95% CI: 0.31–0.39) (Table 1 and Figures 1–3).

### Mortality and DALYs trends

The number of liver cancer deaths in China rose from 94,937 (95% CI: 79,884 – 111,527) in 1990 to 172,068 (95% CI: 139,621 – 212,496) in 2021. Concurrently, the ASMR decreased from 10.75 per 100,000 (95% CI: 9.12–12.61) to 8.35 per 100,000 (95% CI: 6.80–10.29), with an AAPC of −0.79% (95% CI: −1.18 to −0.41). In contrast, global liver cancer deaths increased from 238,969 (95% CI: 218,717 – 263,037) in 1990 to 483,875 (95% CI: 440,400 – 540,177) in 2021, while the ASMR remained relatively stable, with a slightly decline from 5.86 per 100,000 (95% CI: 5.38–6.46) to 5.65 per 100,000(95% CI: 5.13–6.30), reflected by an AAPC of −0.11% (95% CI: −0.23 – 0.01) (Table 1 and Figures 1–3).

The DALY attributed to liver cancer in China escalated from 3,294,864 (95% CI: 2,763,029 – 3,879,589) in 1990 to 4,890,023 (95% CI: 3,905,089 – 6,124,599) in 2021. Nonetheless, the ASDR significantly decreased from 334.52 per 100,000 population (95% CI: 281.08–393.14) to 239.91 per 100,000 population (95% CI: 191.98–299.37), resulting in an AAPC of −1.03% (95% CI: −1.32 to −0.74). Globally, DALYs increased from 7,553,667 (95% CI: 6,897,511 – 8,296,182) in 1990 to 12,887,652 (95% CI: 11,673,533 – 14,472,228) in 2021, while the ASDR showed a decline from 172.86 per 100,000 (95% CI: 157.84–190.16) to 149.29 per 100,000 (95% CI: 135.24–167.49), reflected by an AAPC of −0.46% (95% CI: −0.67 – −0.26) (Table 1 and Figures 1–3).

### Comparative trends in ASIR, ASMR, and ASDR

China exhibited a pronounced decline in the ASIR, ASMR, and ASDR for liver cancer compared to global averages. The ASIR decreased from 10.58 to 9.52 per 100,000 in China, while globally, it slightly increased from 5.90 to 6.15 per 100,000. Both the ASMR and ASDR showed greater reductions in China than in global trends, emphasizing the effectiveness of public health measures (Table 1 and Figures 1–3).

### Recent trends (2019–2021)

Between 2019 and 2021, the number of liver cancer cases in China decreased from 198,004 to 196,637, while the ASIR declined from 9.98 to 9.52 per 100,000. Among males, cases dropped from 145,125 to 143,788, and the ASIR fell from 14.94 to 14.34 per 100,000. For females, the number of cases remained stable, from 52,878 to 52,848, with the ASIR decreasing from 5.19 to 4.89 per 100,000. Globally, liver cancer cases increased from 512,927 in 2019 to 529,202 in 2021, although the ASIR decreased from 6.24 to 6.15 per 100,000. Among males, cases rose from 354,003 to 364,354, but the ASIR decreased from 9.09 to 8.98 per 100,000. Among females, cases increased from 158,924 to 164,848, though the ASIR dropped from 3.64 to 3.60 per 100,000 (Table 2).

**Table 2:** All-age cases and age-standardized incidence, prevalence, mortality, and DALY rates for different sexes of liver cancer in China and globally in 2019 and 2021.

Location	Measure	Both		Male		Female	
		Number	ASR (1/100,000)	Number	ASR (1/100,000)	Number	ASR (1/100,000)
China (2019)	Incidence	198,004	9.98	145,125	14.94	52,878	5.19
	Prevalence	268,811	13.87	201,372	20.78	67,439	7.02
	Deaths	173,701	8.83	124,030	13.03	49,671	4.88
	DALYs	5,025,364	253.71	3,815,466	388.16	1,209,899	119.57
Global (2019)	Incidence	512,927	6.24	354,003	9.09	158,924	3.64
	Prevalence	721,919	8.82	508,425	12.94	213,493	5.05
	Deaths	468,410	5.72	315,197	8.2	153,213	3.5
	DALYs	12,644,770	152.5	8,936,219	222.48	3,708,551	86.47
China (2021)	Incidence	196,637	9.52	143,788	14.34	52,848	4.89
	Prevalence	265,539	13.29	198,826	20	66,713	6.64
	Deaths	172,068	8.35	122,463	12.4	49,605	4.57
	DALYs	4,890,023	239.91	3,702,093	368.19	1,187,930	111.91
Global (2021)	Incidence	529,202	6.15	364,354	8.98	164,848	3.6
	Prevalence	739,300	8.68	520,567	12.76	218,732	4.95
	Deaths	483,875	5.65	324,696	8.1	159,179	3.46
	DALYs	12,887,652	149.29	9,076,177	217.65	3,811,476	85.06

From 2019 to 2021, liver cancer deaths in China fell slightly from 173,701 to 172,068, with a decrease in ASMR from 8.83 to 8.35 per 100,000. Among males, deaths decreased from 124,030 to 122,463, and the ASMR dropped from 13.03 to 12.40 per 100,000. For females, deaths remained stable, while the ASMR declined from 4.88 to 4.57 per 100,000. Globally, liver cancer deaths rose from 468,410 in 2019 to 483,875 in 2021, despite a slight decline in the global ASMR from 5.72 to 5.65 per 100,000 (Table 2).

### Gender and age patterns

**Figure 4:**
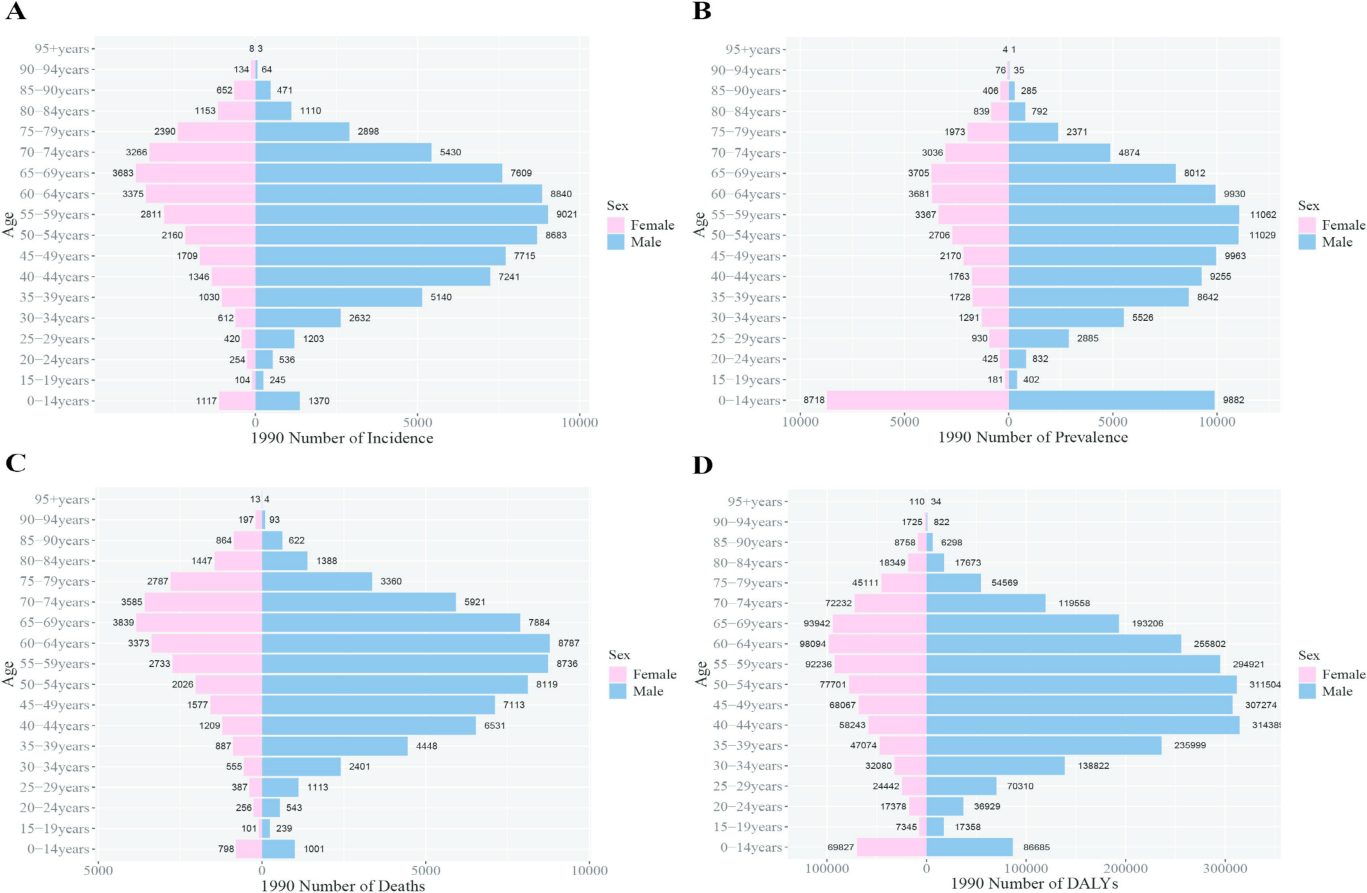
Comparison of the incidence, prevalence, mortality, and DALYs of liver cancer in males and females in different age groups in China in 1990. **(A)** Incidence; **(B)** prevalence; **(C)** mortality; **(D)** DALYs.

**Figure 5:**
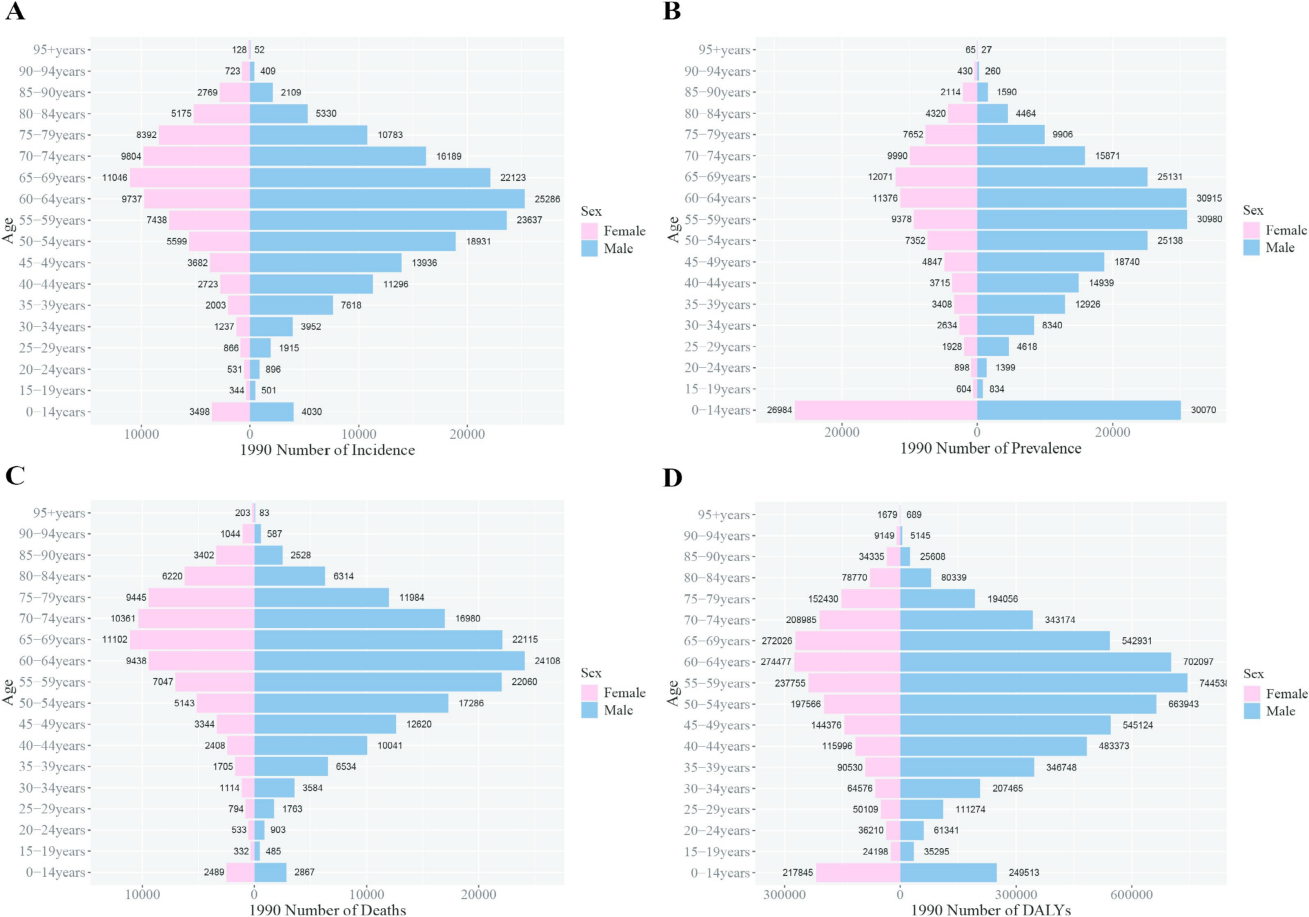
Comparison of the incidence, prevalence, mortality, and DALYs of liver cancer in males and females in different age groups worldwide in 1990. **(A)** Incidence; **(B)** prevalence; **(C)** mortality; **(D)** DALYs.

In 1990, the peak incidence and prevalence rates for liver cancer among Chinese males occurred at ages 55–59, whereas for females, the peaks were observed at ages 65–69. The peak mortality rate for males was at ages 60–64, while for females, it was at ages 65–69. Notably, the peak DALY rate for Chinese males occurred earlier, at ages 40–44, compared to the global peak at ages 55–59, signifying a 15-year earlier burden peak for Chinese males. For females, both in China and globally, the peak DALY rate was observed at ages 60–64 (Figures 4, 5).

**Figure 6:**
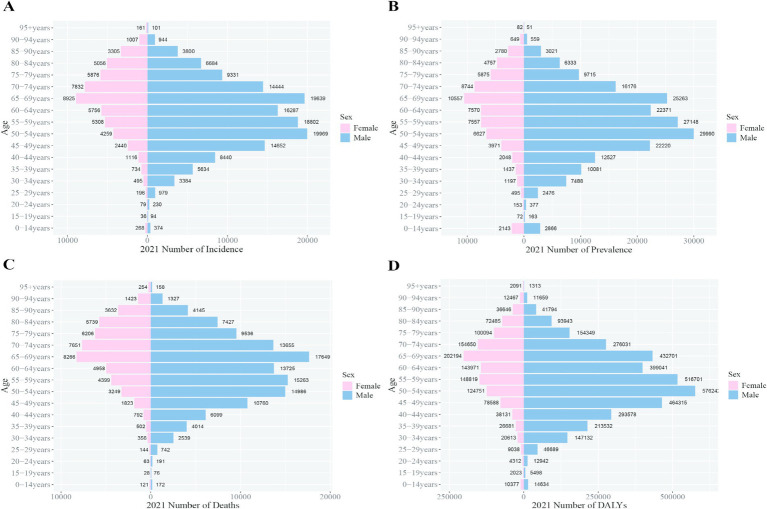
Comparison of the incidence, prevalence, mortality, and DALYs of liver cancer in males and females in different age groups in China in 2021. **(A)** Incidence; **(B)** prevalence; **(C)** mortality; **(D)** DALYs.

**Figure 7:**
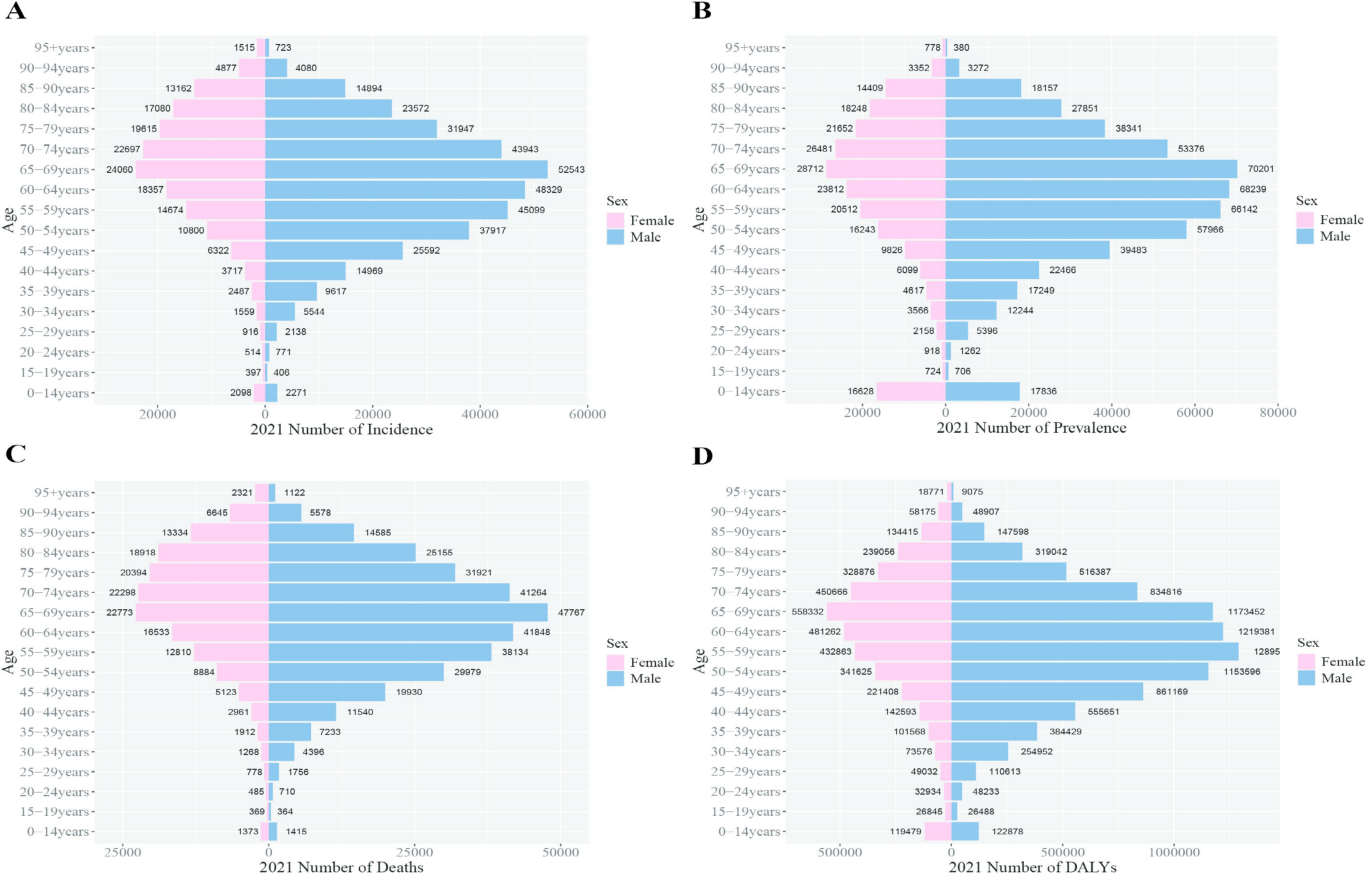
Comparison of the incidence, prevalence, mortality, and DALY rates of liver cancer in males and females in different age groups worldwide in 2021. **(A)** Incidence; **(B)** prevalence; **(C)** mortality; **(D)** DALYs.

By 2021, the peak incidence, prevalence, and DALY rates for Chinese males shifted to ages 50–54, while for females, the peak persisted at ages 65–69. The peak mortality rate for both sexes in China was recorded at ages 65–69. Globally, the peak incidence, prevalence, and mortality rates for both sexes occurred at ages 65–69. The global DALY rate for males peaked at ages 55–59, whereas for females, the peak remained at ages 65–69. These trends indicate that the burden peak for Chinese males occurred 5 years earlier than the global average (Figures 6, 7).

**Figure 8:**
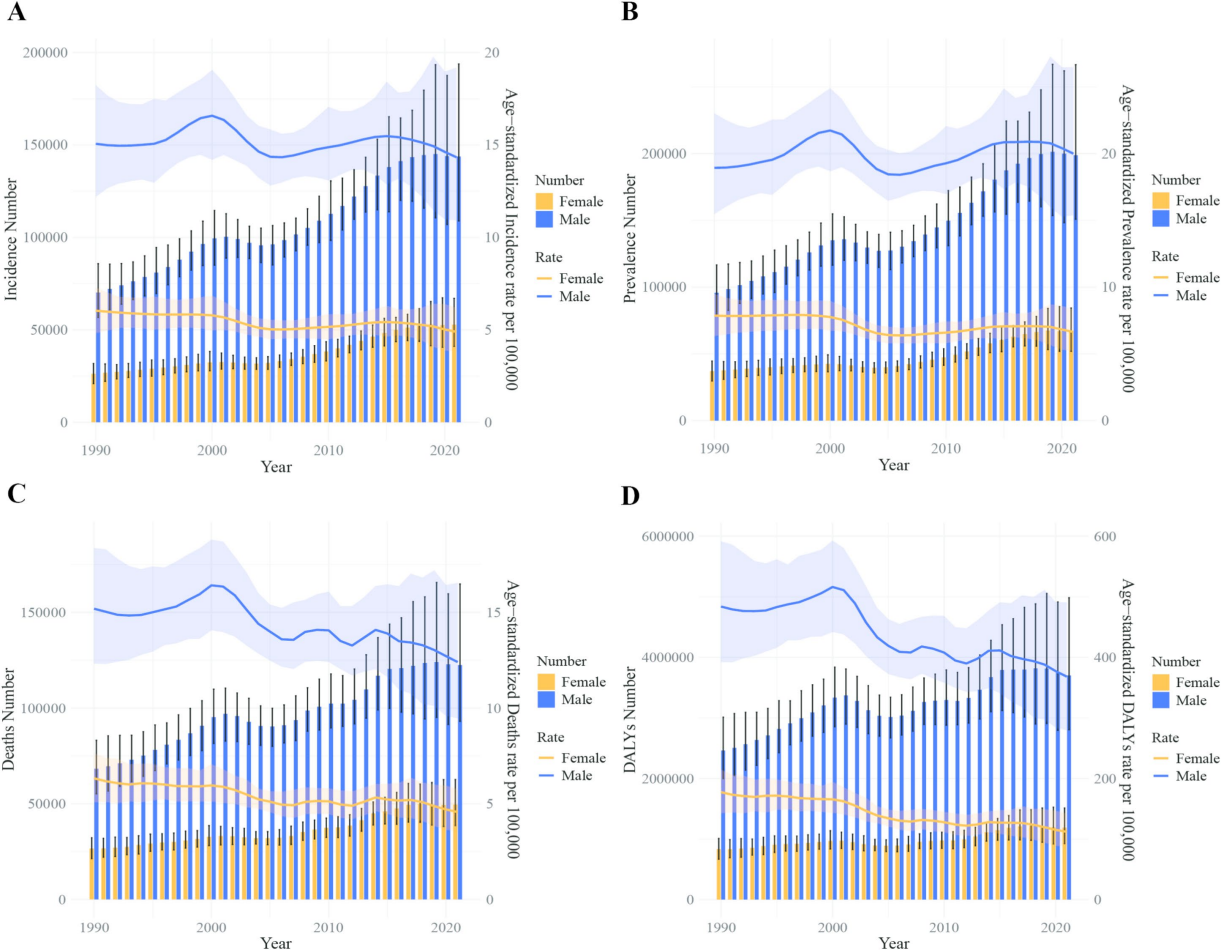
Comparison of full-age cases and age-standardized rates of incidence, prevalence, mortality and DALYs among men and women in China from 1990 to 2021. **(A)** Incident cases and ASIR; **(B)** prevalent cases and ASPR; **(C)** death cases and ASMR; **(D)** DALY counts and ASDR. Bar charts represent counts; lines represent age-standardized rats.

**Figure 9:**
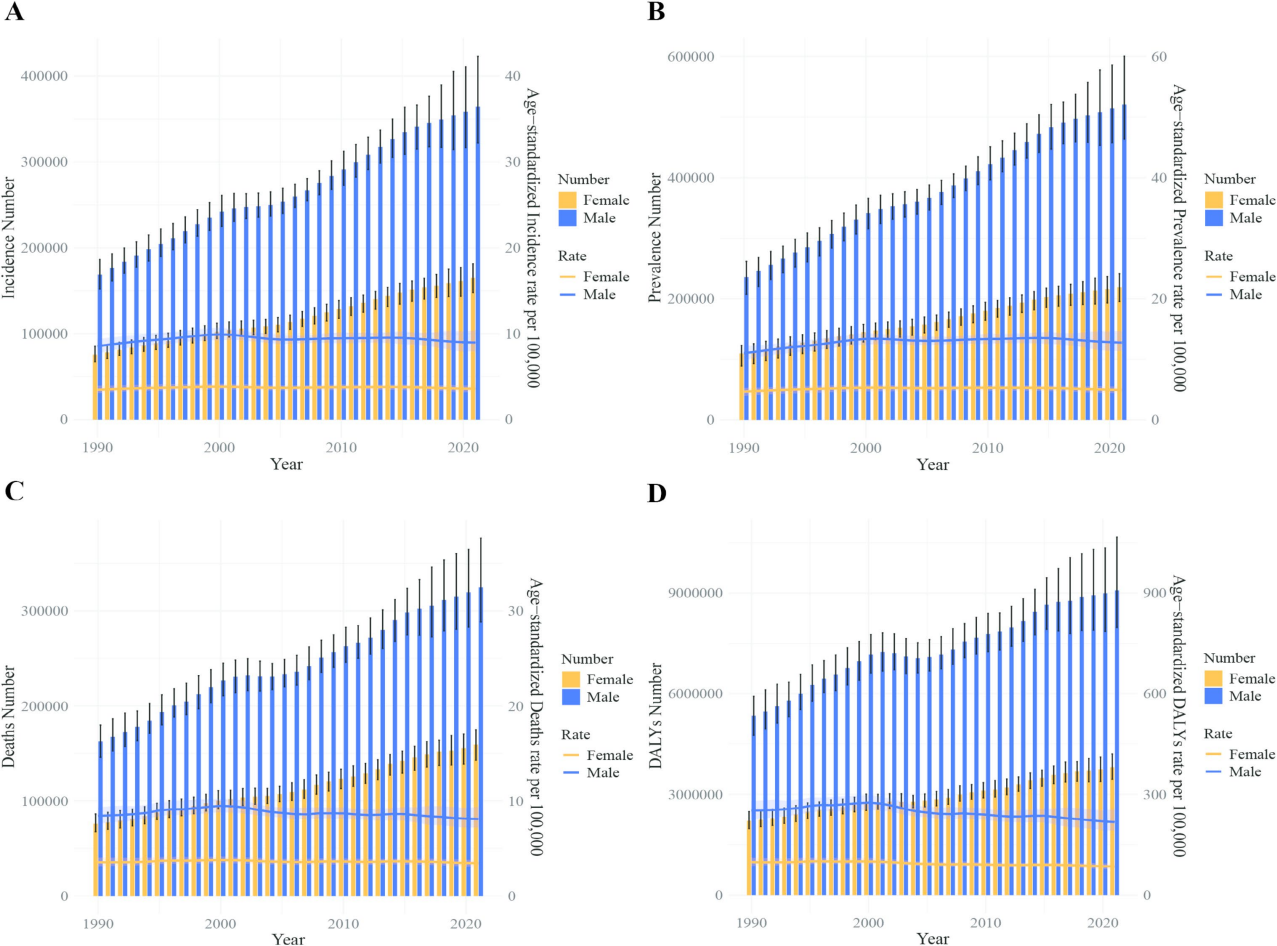
Comparison of full-age cases and age-standardized rates of incidence, prevalence, mortality and DALYs among men and women worldwide from 1990 to 2021. **(A)** Incident cases and ASIR; **(B)** prevalent cases and ASPR; **(C)** death cases and ASMR; **(D)** DALY counts and ASDR. Bar charts represent counts; lines represent age-standardized rates.

The number of DALYs due to liver cancer in China decreased from 5,025,364 to 4,890,023, with the ASDR dropping from 253.71 to 239.91 per 100,000 people. Among males, DALYs fell from 3,815,466 to 3,702,093, while the ASDR decreased from 388.16 to 368.19 per 100,000. Among females, DALYs dropped from 1,209,899 to 1,187,930, with the ASDR declining from 119.57 to 111.91 per 100,000. Globally, DALYs increased from 12,644,770 in 2019 to 12,887,652 in 2021, while the ASDR decreased from 152.50 to 149.29 per 100,000 people. For males, DALYs rose from 8,936,219 to 9,076,177, but the ASDR dropped from 222.48 to 217.65 per 100,000. Among females, DALYs increased from 3,708,551 to 3,811,476, while the ASDR decreased from 86.47 to 85.06 per 100,000 (Figures 8, 9).

## Discussion

Based on data from the GBD 2021 database, this study offers a comprehensive evaluation of the incidence, prevalence, mortality, and DALYs related to liver cancer in China and globally from 1990 to 2021. The analysis primarily focuses on comparing disparities in liver cancer burden across various ages and sexes. Our findings reveal that between 1990 and 2021, China’s ASIR, ASMR, and ASDR for liver cancer have exhibited a notable decline. However, the ASPR has remained relatively stable. In contrast, the global trends display a more varied pattern, with a rise in the ASIR and ASPR, while the ASMR has remained relatively stable, and the ASDR has shown a slight decline.

Notably, the ASDR ratio of China to the global average decreased from 1.94 (334.52/172.86) in 1990 to 1.61 (239.91/149.29) in 2021, indicating a narrowing gap between China and the global liver cancer burden. Despite this progress, China continues to experience a higher burden than the global average. These results align with previous studies, which have also highlighted a significant reduction in the ASPR of liver cancer in Asia, particularly in countries like China that have implemented successful hepatitis B virus (HBV) vaccination programs and other public health interventions (19–21). Meanwhile, other regions, including Europe, the Americas, and Africa, have seen an increase in liver cancer prevalence. The United States, in particular, has experienced the highest ASPR increase. Such regional disparities underscore the influence of various risk factors on liver cancer incidence and highlight the stark differences in trends between high- and low-income countries.

High-income countries like the United States tend to experience increased liver cancer rates due to factors such as a higher prevalence of hepatitis C virus (HCV) infections, alcohol consumption, and the ability to conduct more comprehensive cancer diagnostics (22), studies have found that the large-scale refugee crisis in Europe has significantly altered the disease burden of HBV and HCV, thereby influencing the disease burden of liver cancer (23). Conversely, low-income countries, including China, have historically faced a disproportionate burden from HBV-associated liver cancer. However, the implementation of extensive vaccination campaigns has steadily reduced the incidence of liver cancer in these regions (24). Despite this, challenges such as limited healthcare access, diagnostic delays, and insufficient treatment options remain significant barriers (25). Additionally, socioeconomic factors such as education, income, and living conditions further contribute to the discrepancies in liver cancer burden, especially older men with obesity, alcohol consumption, tobacco use, and drug abuse remain high-risk factors for the development of liver cancer, with metabolic-dysfunction associated steatotic liver disease (MASLD) are increasing globally, emphasizing the need for targeted public health strategies to address these problems (26).

The COVID-19 pandemic, which emerged in 2019, increased the global burden of diseases by 4.1% in 2020 and 7.2% in 2021, respectively, resulting in over 830,000 liver cancer-related deaths in 2020 (20). However, interestingly, our study revealed that in 2021, both in China and globally, the ASIR, ASPR, ASMR, and ASDR of liver cancer were lower than the levels reported in 2019, indicating that the COVID-19 pandemic did not directly exacerbate the liver cancer burden. This finding is consistent with global research outcomes (10), but we cannot ignore the potential impact of the COVID-19 pandemic on liver cancer burden, such as disruptions in healthcare services and screening programs led to the false impression that the burden of liver cancer has not increased.

Furthermore, our results align with existing studies demonstrating that sex plays a crucial role in the liver cancer burden, with males consistently exhibiting higher incidence and mortality rates compared to females. This disparity is primarily driven by differential exposure to key risk factors such as HBV infection and alcohol consumption, which are more prevalent among men (27–29). Additionally, studies have shown that differences in sex hormones are associated with the higher disease burden of liver cancer in men compared to women, the hypothesis that greater capacity to convert dehydroepiandrosterone (DHEA) could be associated with increased liver cancer risk among men (30). The earlier peak burden of liver cancer in Chinese males, observed 5 years before the global average, suggests the need for more targeted preventive efforts, particularly those aimed at managing risk factors such as viral hepatitis, obesity, diabetes, drug abuse, alcohol use, and tobacco consumption (31–36).

Over the past three decades, China has made significant strides in reducing the burden of liver cancer, as demonstrated by the declining trends in the ASIR, ASMR, and ASDR. The decrease in liver cancer burden in China is more pronounced compared to global trends, largely due to successful public health interventions, such as widespread hepatitis B vaccination, improved access to healthcare, and advancements in early detection and treatment. However, despite these improvements, China still faces a higher liver cancer burden compared to the global average, particularly in males, who exhibit higher rates of incidence, mortality, and DALYs across all age groups.

The earlier peak of liver cancer burden among Chinese males compared to the global average highlights the need for more targeted prevention and control strategies. Risk factors such as hepatitis B and C infections, obesity, diabetes, drug abuse, alcohol consumption, and tobacco use continue to play significant roles in liver cancer development, particularly in China. As such, strengthening efforts in vaccination, screening, and lifestyle interventions is critical for further reducing the liver cancer burden in the country.

On April 2, 2025, the National Patriotic Health Campaign Committee issued the “Notice of the National Patriotic Health Campaign Committee on Incorporating Three Actions, Including the Healthy Weight Management Action, into the Healthy China Initiative.” The implementation of this national policy will have a positive impact on preventing obesity and, in turn, reducing the disease burden of conditions such as liver cancer, which are associated with increased risk factors caused by obesity (37).

Globally, liver cancer remains a significant public health challenge, with rising incidence and prevalence rates, especially in regions with historically low burdens, such as the United States and European region. This increase is likely driven by lifestyle factors, including alcohol and tobacco consumption, obesity, hepatitis C infection, refugee crisis, emphasizing the need for a comprehensive global approach to liver cancer prevention. For example, similar to China, it could implement universal hepatitis vaccination, manage weight to avoid obesity, promote healthy lifestyles, and formulate reasonable refugee policies.

## Conclusion

In conclusion, while China has made considerable progress in reducing the liver cancer burden, continued efforts are required to address the remaining challenges, particularly in the areas of risk factor management and healthcare access. Similarly, global efforts must focus on prevention strategies that target high-risk populations and regions experiencing an increasing liver cancer burden. Future research should aim to monitor emerging trends, develop novel prevention strategies, and improve early detection and treatment methods to achieve further progress in combating this deadly disease.

## Data Availability

The raw data supporting the conclusions of this article will be made available by the authors, without undue reservation.
